# Sex-specific differences in the efficacy of renal denervation in patients with resistant hypertension depending on visceral obesity and kidney function

**DOI:** 10.3389/fcvm.2025.1501296

**Published:** 2025-01-30

**Authors:** Irina Zyubanova, Nadezhda Ryumshina, Victor Mordovin, Musheg Manukyan, Valeriya Lichikaki, Ekaterina Solonskaya, Anna Gusakova, Tatjana Suslova, Stanislav Pekarskiy, Simzhit Khunkhinova, Anastasia Popova, Veronika Rudenko, Alla Falkovskaya

**Affiliations:** Hypertension Department, Cardiology Research Institute, Tomsk National Research Medical Center, Russian Academy of Sciences, Tomsk, Russia

**Keywords:** renal denervation, resistant hypertension, responders, visceral obesity, perirenal fat, renal function, chronic kidney disease

## Abstract

**Objective:**

To investigate the sex differences in the efficacy of renal denervation (RDN) in patients with resistant hypertension (RHT) concerning the size of abdominal fat depots, changes in biomarkers of sympathetic activity, and renal function.

**Materials and methods:**

24 men (56.5 ± 7.8 years) and 33 women (59.5 ± 8.4 years) with RHT were enrolled in the study and underwent RDN. 24-h ambulatory blood pressure (BP) [systolic/diastolic (SBP/DBP)], serum creatinine (with eGFR calculation), serum adipocytokines (leptin, adiponectin, resistin), serum metanephrines and normetanephrines were measured baseline and 12 months after RDN. The size of subcutaneous, visceral, and perirenal adipose tissue (SAT, VAT, and PRAT) was assessed using MRI.

**Results:**

After RDN, BP decreased, leptin increased, and adiponectin, resistin, and metanephrine levels did not change in both sexes. There was a decrease in normetanephrine levels in women and a similar trend in men. In men, the eGFR did not change. In women, the eGFR remained unchanged only in those with chronic kidney disease (CKD) (*n* = 10) and decreased in the absence of CKD (*n* = 23) from 79.7 ± 14.1 to 72.1 ± 12.0 ml/min/1.73 m^2^ (*p* = 0.011). Men had larger visceral fat depots, and women had larger subcutaneous fat depots. After RDN, the size of adipose tissue in men remained unchanged, and in women, the PRAT thickness decreased from 2.36 ± 1.23 to 2.10 ± 1.17 cm (*p* = 0.002). Lowering BP in women was associated with increased leptin levels after RDN (*r* = −0.47 for SBP, *r* = −0.48 for DBP). Dependence of BP reduction on baseline eGFR was observed in men only [*r* = 0.44 for SBP, *r* = 0.48 for pulse pressure (PP)]. Additionally, in men, the decrease in SBP and PP depended on VAT areas (*r* = −0.44 and *r* = −0.58, respectively). In women, the SBP reduction showed an inverse correlation between baseline weight (*r* = −0.35) and waist circumference (*r* = −0.38).

**Conclusions:**

The magnitude of the antihypertensive effect of RDN depends on signs of visceral obesity and, in men, also on the presence of CKD. Renoprotective effects of RDN in men are obtained regardless of the initial kidney function, while in women, it was observed only in individuals with CKD. Additional beneficial effects of RDN in women include a decrease in normetanephrine levels and a reduction in PRAT size.

## Introduction

Hypertension (HT) is one of the main causes of cardiovascular disease and complications such as stroke, coronary artery disease, and heart failure (1), as well as severe kidney damage (2). Treatment-resistant hypertension (RHT) is identified when blood pressure (BP) remains above 140/90 mmHg despite recommended lifestyle changes and the use of three classes of antihypertensive medication, including a diuretic (3).

Catheter-based radiofrequency renal denervation (RDN) is a minimally invasive procedure that ablates the periarterial renal nerve fibers and reduces sympathetic efferent signaling to the kidneys and sensory afferent signaling from the kidneys. In numerous previous clinical trials, RDN has demonstrated its efficacy in lowering blood pressure and its safety (4). In the 2023 European Society of Hypertension Guidelines, RDN is recommended for the treatment of hypertension (5).

RDN has been shown to be equally effective in patients with and without chronic kidney disease (CKD) (6) and also preserves kidney function in both patient groups (7, 8). RHT and CKD are pathogenetically linked. In CKD, sodium retention and volume expansion are the main causes of hypertension. However, other factors, such as activation of the renin-angiotensin-aldosterone system (RAAS) and overactivity of the sympathetic nervous system (SNS), are additional pathogenetic mechanisms that promote hypertension (9). Increased body weight and obesity are important risk factors for both diseases—RHT and CKD—and are common in both.

Mechanisms that trigger HT in obesity include increased extracellular fluid volume and increased venous return and cardiac output, activation of the SNS and RAAS, meta-inflammation, and mechanical compression of the kidneys by excessive growth of perinephric fat (10). On the other hand, obesity is a driving force for the progression of CKD, and mechanisms also include hemodynamic changes, metainflammation, and activation of the SNS and RAAS (11).

Obesity-related hypertension should be responsive to sympathetic RDN as it is directly related to increased SNS activity, particularly renal sympathetic outflow. However, the data on this subject are absolutely contradictory: a number of studies confirm the greater efficacy of RDN in obese patients (12), while a number confirm it in normal body weight (13). The SYMPLICITY-3 study found no correlation between body mass index and response (14).

It is clear that the efficacy of renal denervation is limited. It has been reported that approximately 30% of patients did not respond to treatment after the procedure [defined as a reduction in systolic blood pressure (SBP) of <5 mmHg for ambulatory or 10 mmHg for office SBP], and in some patients, SBP even increased (15). In general, blood pressure decreased more after RDN in patients who had higher blood pressure before the procedure (16). In addition, there is evidence that younger people (17), patients with increased blood pressure variability (18), obstructive sleep apnea (19), and less aortic calcification (20) respond better to RDN in terms of blood pressure reduction. In the RADIANCE-HTN SOLO trial, overweight female patients responded best to the procedure (21).

Abdominal and non-abdominal obesity essentially correspond to visceral and subcutaneous fat (22), and this classification is based on the anatomical and physiological characteristics of fat depots. When we talk about the negative effects of obesity, we essentially mean visceral obesity. In fact, visceral obesity is a key component of metabolic syndrome and an important risk factor for kidney and cardiovascular disease (CVD) (23). Perirenal fat refers to visceral adipose tissue and is a fat pad in the retroperitoneal space surrounding the kidneys. Some studies have shown that perirenal fat is an independent risk predictor for CVD (24). Perirenal fat is located near the kidneys; it is active in metabolism and in the secretion of adipokines, which accelerate the processes of renal damage and can also cause the development of RHT.

It is known that the size of fat depots differs significantly in men and women, with visceral fat predominant in men and subcutaneous fat in women. It is clear that body mass index (BMI) is not an accurate indicator of obesity and that more specific investigations are required to assess visceral obesity, such as magnetic resonance imaging (MRI), computed tomography, and ultrasound. It remains unclear to what extent the size of visceral fat depots in men and women with RHT is related to blood pressure and renal function and whether the efficacy of renal denervation depends on the degree of abdominal obesity. We hypothesize that if adipose tissue in the abdominal and perinephral region may affect blood pressure and renal function when excessively accumulated, in part due to overactivity of the sympathetic nervous system, the results of the sympatholytic procedure—renal denervation—may also depend on the size of these fat depots. At the same time, the value of subcutaneous and visceral fat, as well as the imbalance of adipokines and renal function, may differ significantly between men and women.

The aim was to investigate the sex differences in the efficacy of renal denervation in patients with resistant hypertension concerning the size of abdominal fat depots, changes in biomarkers of sympathetic activity, and renal function.

## Materials and methods

The study population included 24 men and 33 women with uncontrolled RHT who were comparable in age, percentage of obesity, and 24-h SBP and underwent RDN at the Research Institute of Cardiology of the Tomsk National Medical Research Center from September 2012 to February 2021. Patients were receiving three or more antihypertensive medications, including diuretics, which had not been changed in the three months prior to enrollment in the study. RHT was assessed according to the European Society of Cardiology and European Society of Hypertension (ESC/ESH) guidelines for the treatment of HT (25).

Exclusion criteria were pseudoresistant or secondary HT, an estimated glomerular filtration rate (eGFR) <30 ml/min/1.73 m^2^, inflammatory disease and renal tumors, pregnancy, renal artery anatomy ineligible for treatment, a pacemaker incompatible with MRI, severe concomitant diseases or other diseases that did not allow an MRI examination. Information on drug treatment was obtained through a questionnaire.

The study was conducted in accordance with the applicable national and international standards (Good Clinical Practice and the principles of the Declaration of Helsinki). The local ethics committee approved the study protocol (excerpt from the biomedical ethics committee meeting #60 min of March 02, 2010 and #210 min of February18, 2021). All patients gave written informed consent before participating in the study. The clinical characteristics of the patients are shown in Table 1.

**Table 1:** Clinical characteristic [M ± SD, *n* (%)].

Parameters	Women (*n* = 33)	Men (*n* = 24)	*p*
Age, years	59.5 ± 8.4	56.5 ± 7.8	0.159
BMI, g/m^2^	36.0 ± 4.7	32.4 ± 4.1	0.004
Weight, kg	90.8 ± 11.4	100.6 ± 13.4	0.004
WC, cm	107.8 ± 12.0	107.0 ± 10.7	0.859
Obesity, *n* (%)	29 (88%)	18 (75%)	0.694
Abdominal obesity, *n* (%)	31 (94%)	18 (75%)	0.573
Coronary artery disease, *n* (%)	23 (70%)	10 (42%)	0.266
Chronic kidney disease, *n* (%)	10 (30%)	5 (20%)	0.538
eGFR, ml/min/1.73 m^2^	69.8 ± 19,9	76.7 ± 17,6	0.022
Diabetes mellitus type 2, *n* (%)	19 (58%)	9 (37.5%)	0.376
24-SBP, mm Hg	161.3 ± 19,9	156.8 ± 10.7	0.322
24-DBP, mm Hg	83.6 ± 14.6	91.1 ± 11.3	0.040
24-PP, mm Hg	77.7 ± 18.4	65.8 ± 10.6	0.006
No antihypertensive drugs	4.5 ± 1.0	4.4 ± 1.2	0.790
Catheter Symplicity flex/spyral	24/9	14/10	0.424

Data are presented as number (%), mean ± standard deviation. BMI, body mass index; WC, waist circumference; eGFR, estimated glomerular filtration rate; SBP, systolic blood pressure; DBP, diastolic blood pressure; PP, pulse pressure.

Although women had a higher BMI, the percentage of obese individuals did not differ between men and women. The same was true for eGFR. The percentage of patients with CKD did not differ (CKD was defined as eGFR <60 ml/min/1.73 m^2^). But at the same time, men had higher diastolic BP (DBP) and correspondingly lower pulse BP (PP) levels.

Before and 12 months after renal denervation, in addition to standard clinical examination (body weight, BMI, waist circumference (WC), creatinine with assessment of renal function (using eGFR, CKD-EPI formula), 24-h ambulatory BP monitoring (ABPM), blood sampling for biomarkers and MRI of the abdominal area were performed.

Based on the oscillometric method, ABPM was performed using an automated ABPM-04 system (Meditech, Hungary).

Blood samples were taken from the antecubital vein on an empty stomach in the morning. The levels of leptin, adiponectin, and resistin in serum were determined with kits from Mediagnost (Germany); metanephrines and normetanephrines were determined with kits from IBL International GmbH (Germany). The ranges of the reference values of the biochemical markers are listed in Table 2.

**Table 2:** Reference values of the biochemical markers.

Marker	Reference values
Leptin	3.7–11.1 ng/ml (female)
	2.0–5.6 ng/ml (male)
Adiponectin	8.2–19.1 μg/ml
Resistin	7.41 ± 2.47 ng/ml (female)
	6.48 ± 2.44 ng/ml (male)
Metanephrines	Less than 90 ng/ml
Normetanephrines	Less than 190 ng/ml

MRI was performed on a tomograph with a magnetic field induction of 1.5 T (Titan Vantage, Toshiba, 2010) using eight-channel receiving coils for the whole body, with the patient lying supine and the arms placed along the body with synchronization by breathing. Coronal and axial T1- and T2-weighted (TSE) and fat saturation (FS) images were acquired.

The scan area covered the distance from the dome of the diaphragm to the pelvic inlet. Image processing was performed using 3D Slicer 4.9.0 software (2018) and Centricity Universal Viewer v.6.0 (GE, 2020) based on the Medical Genomics Shared Use Center. The delineation of adipose tissue was performed semi-automatically, according to the selected area of MR signal intensity corresponding to the adipose tissue. If necessary, areas that were within the intensity range (intestinal contents, renal collecting system, vessels) were manually excised.

All MRI scans were guided and interpreted by a single experienced examiner who was blinded to the results of the RDN.

The following indicators were determined:

- Visceral adipose tissue area (VATa) and subcutaneous adipose tissue area (SATa) were measured separately on axial T2-weighted images at the level of the L4–L5 intervertebral disc. Area calculations were performed automatically using software based on the number of voxels included in the MR signal intensity range;
- Kidney diameter was measured on axial T1-FS images at the level of the renal vein as the anteroposterior diameter of the kidney;
- Perirenal adipose tissue (PRAT) thickness was measured as the difference between the Gerota's diameter and the kidney diameter; Gerota's diameter was measured as the distance between the layers of Gerota's fascia;
- Anterior subcutaneous fat thickness (SATth) was measured on axial T2-weighted images at the level of the umbilicus.

RDN interventions were performed using Symplicity Flex (*n* = 38) and Symplicity Spyral (*n* = 19) catheters according to the manufacturer's instructions, with treatments performed mainly on the distal branches of the renal artery, as we have previously described (26).

Patients with a reduction of 5 mmHg or more in ambulatory SBP after the procedure were classified as responders.

The statistical analysis was carried out using Statistica v.10.0 software. The Shapiro–Wilk test was used to test the hypothesis of normal distribution of continuous variables. Results were presented as mean with standard deviation (±SD) when data were parametrically distributed and as median with interquartile range (Q1;Q3). Differences between groups were tested with unpaired *t*-tests for normally distributed variables and otherwise with the Mann–Whitney *U*-test. The paired *t*-test or the Wilcoxon signed-rank test determined within-group differences in repeated measures. A contingency table analysis (Pearson chi-square) was used to analyze the qualitative data. The relationship between the variables was assessed using Spearman's correlation coefficient (*r*). The statistical significance was set at *p* < 0.05. The delta (Δ) of the parameter was calculated as the difference between the value obtained after 12 months and a similar value.

## Results

### Changes in blood pressure after RDN

SBP, DBP, and PP decreased significantly after renal artery denervation in both men and women (Table 3).

**Table 3:** Changes in blood pressure between baseline and 12 months after RDN in men and women [M ± SD].

Parameter	Men (*n* = 24) 19 responders (79%)			Women (*n* = 33) 23 responders (70%)		
	Baseline	12 month	*р*	Baseline	12 month	*р*
24-SBP, mm Hg	156.8 ± 10.7	144.3 ± 15.5	0.001	161.3 ± 19.9	144.9 ± 13.3	0.0001
24-DBP, mm Hg	91.1 ± 11.3	84.5 ± 11.1	0.002	83.6 ± 14.6	75.8 ± 12.9	0.0006
24-PP, mm Hg	65.8 ± 10.6	59.7 ± 12.7	0.006	77.7 ± 18.4	59.2 ± 14.8	0.001

Data are presented as mean ± standard deviation. SBP, systolic blood pressure; DBP, diastolic blood pressure; PP, pulse pressure.

### Biomarkers

Initially, leptin levels exceeded the normal range, particularly in female patients. However, the levels of the other adipokines were within the reference values and showed no significant differences between the sexes. The majority of men (92%) and women (94%) with RHT exhibited blood normetanephrine levels above the normal range; metanephrines exceeded the normal range in 30% of men and 50% of women; there were no significant sex differences in these indicators (Table 4).

**Table 4:** Baseline values of biochemical markers in men and women [Me(Q1;Q3)].

Parameters	Women (*n* = 33)	Men (*n* = 24)	*p*
Leptin, ng/ml	51.3[40.4;70.7]	15.6[11.7;22.3]	<0.0001
Adiponectin, μg/ml	6.16[4.45;8.36]	5.42[4.38;8.42]	0.667
Resistin, ng/ml	5.07[4.10;6.71]	4.63[3.87;5.66]	0.428
Metanephrines, ng/ml	90.2[61.6;135.4]	59.8[51.9;96.2]	0.246
Normetanephrines, ng/ml	297.4[214.2;523.5]	277.9[180.8;334.3]	0.392

The data are presented as median with interquartile range.

In both men and women, leptin levels significantly increased after RDN, whereas the levels of other adipokines and metanephrine remained unchanged. A significant decrease in blood normetanephrine levels was observed only in women, with a similar trend noted in men (Table 5).

**Table 5:** Changes in plasma concentrations of biomarkers between baseline and 12 months after RDN in men and women [Me(Q1;Q3)].

Parameter	Men, *n* = 24			Women, *n* = 33		
	Baseline	12 month	*p*	Baseline	12 month	*p*
Leptin, ng/ml	15.6 [11.7;22.3]	22.9 [10.2;29.4]	0.010	51.3 [40.4;70.7]	67.6 [47.4;84.5]	0.011
Adiponectin, μg/ml	5.42 [4.38;8.42]	6.19 [3.57;8.34]	0.554	6.16 [4.45;8.36]	6.68 [4.69;8.80]	0.400
Resistin, ng/ml	4.63 [3.87;5.66]	4.24 [3.78;4.94]	0.435	5.07 [4.10;6.71]	4.68 [3.44;5.90]	0.701
Metanephrines, ng/ml	59.8 [51.9;96.2]	81.2 [77.1;146.8]	0.169	90.2 [61.6;135.4]	88.7 [52.1;103.4]	0.256
Normetanephrines, ng/ml	277.9 [180.8;334.3]	211.5 [182.1;321.8]	0.594	297.4 [214.2;523.5]	210.1 [167.5;314.1]	0.026

Data are presented as median with interquartile range.

### eGFR and CKD

**Figure 1:**
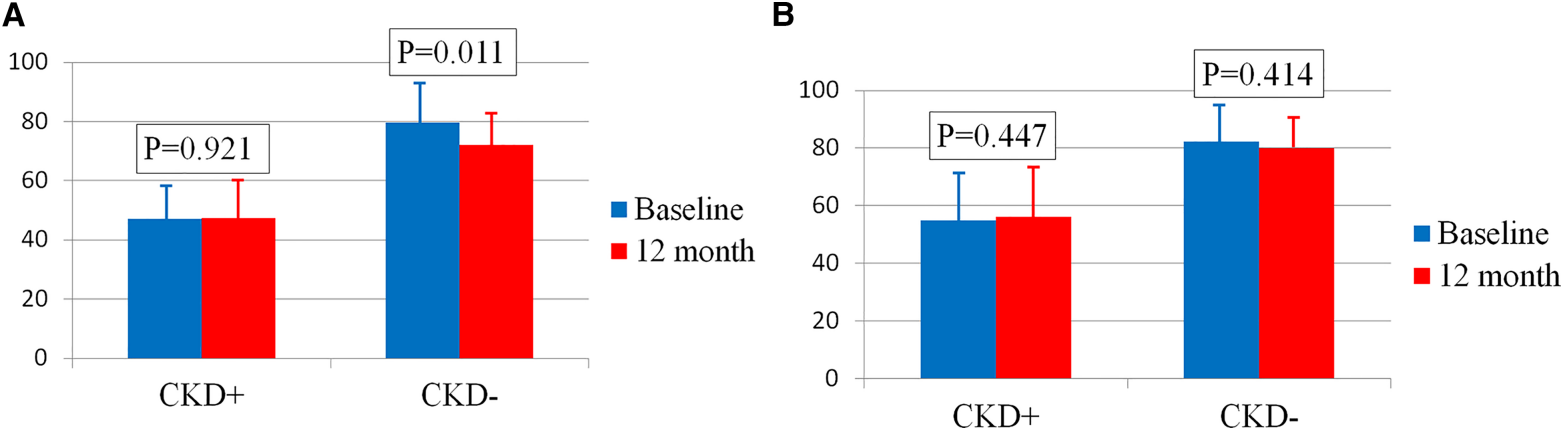
**(A)** Сhanges in eGFR in women with and without CKD. **(B)** Сhanges in eGFR in men with and without CKD. Bar graphs show the respective means ± standard deviations.

In the female group, eGFR significantly decreased after the intervention, from 69.8 ± 19.9 ml/min/1.73 m^2^ to 64.6 ± 16.9 ml/min/1.73 m^2^ (*p* = 0.026). However, closer analysis revealed that this decline occurred exclusively in women without CKD at baseline (*n* = 23) (Figure 1A). In this subgroup, eGFR decreased from 79.7 ± 14.1 ml/min/1.73 m^2^ at baseline to 72.1 ± 12.0 ml/min/1.73 m^2^ at 12 months (*p* = 0.011), remaining above 60 ml/min/1.73 m^2^. Conversely, in women with CKD (*n* = 10), eGFR remained stable, measuring 47.1 ± 10.2 ml/min/1.73 m^2^ at baseline and 47.4 ± 13.0 ml/min/1.73 m^2^ at 12 months (*p* = 0.921).

In men, eGFR remained stable 12 months after RDN, regardless of the presence of CKD (76.7 ± 17.6 ml/min/1.73 m^2^ at baseline vs. 74.9 ± 15.5 ml/min/1.73 m^2^ at 12 months, *p* = 0.478). Among men without CKD (*n* = 19), eGFR was 82.4 ± 12.8 ml/min/1.73 m^2^ at baseline and 79.9 ± 11.0 ml/min/1.73 m^2^ after 12 months (*p* = 0.414). In men with CKD (*n* = 5), eGFR was 54.9 ± 17.6 ml/min/1.73 m^2^ at baseline and 56.2 ± 16.6 ml/min/1.73 m^2^ at 12 months (*p* = 0.447) (Figure 1B).

### Adipose tissue

**Figure 2:**
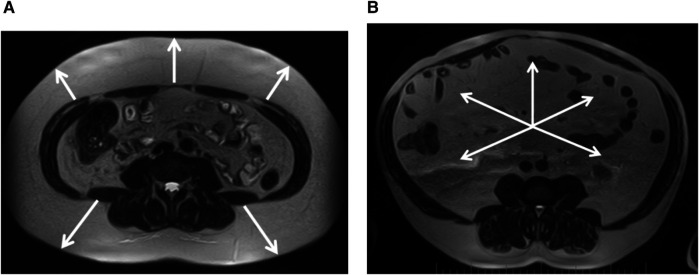
**(A)** Female, 39 years old, pronounced predominance of the subcutaneous adipose tissue (white arrows), VSR = 0.33. **(B)** Male, 57 years old, marked predominance of visceral adipose tissue (white arrows), VSR = 2.21. Magnetic resonance imaging of abdominal adipose tissue/abdominal fat. Axial T2-weighted turbo spin echo images at 4-5 lumbar vertebrae levels.

Sex differences were found in the size of subcutaneous and visceral fat depots in the regions studied. In women, subcutaneous fat was more pronounced (Figure 2A), whereas in contrast, men had larger visceral fat depots (Figure 2B), including PRAT thickness. However, the total visceral adipose tissue area was not significantly dependent on sex in our patients (Table 6).

**Table 6:** Baseline sizes of abdominal and perirenal fat depots in men and women [M ± SD, Me(Q1;Q3)].

Parameters	Women (*n* = 33)	Men (*n* = 24)	*p*
SATa, cm^2^	381.3 ± 114.0	253.8 ± 80.5	0.00002
SATth, cm	3.57 ± 0.83	2.18 ± 0.76	<0.0001
VATa, cm^2^	253.0[176.0;303.0]	270.5[224,5;328.0]	0.136
PRAT, cm	2.36 ± 1.23	3.27 ± 1.35	0.011

Data are presented as mean ± standard deviation or median with interquartile range. SATa, area of subcutaneous adipose tissue; SATth, anterior subcutaneous fat thickness; VATa, area of visceral adipose tissue; PRAT, perirenal adipose tissue.

During 12 months follow-up, the adipose tissue parameters studied remained unchanged in men, while a decrease in PRAT thickness was observed in women. The anthropometric parameters did not change in both groups (Table 7).

**Table 7:** Changes in adipose tissue parameters and anthropometric indicators after RDN [M ± SD, Me(Q1;Q3)].

Parameters	Men (*n* = 24)			Women (*n* = 33)		
	Baseline	12 month	*p*	Baseline	12 month	*p*
SATa, cm^2^	253.8 ± 80.5	251.4 ± 96,6	0.796	381.3 ± 114.0	371.2 ± 102.4	0.438
SATth, cm	2.18 ± 0.76	2.16 ± 0.74	0.726	3.57 ± 0.83	3.62 ± 0.77	0.625
VATa, cm^2^	270.5 [224.5;328.0]	267.0 [211.0;316.5]	0.932	253.0 [176.0;303.0]	247.0 [173.0;290.0]	0.257
PRAT, cm	3.27 ± 1.35	3.29 ± 1.35	0.897	2.36 ± 1.23	2.10 ± 1.17	0.002
BMI, g/m^2^	32.4 ± 4.1	33.2 ± 5.0	0.092	36.0 ± 4.7	35.4 ± 7.5	0.520
Weight, kg	100.6 ± 13.4	101.9 ± 15.7	0.270	90.8 ± 11.4	90.7 ± 12.0	0.871
WC, cm	107.0 ± 10.7	108.9 ± 11.0	0.139	107.8 ± 12.0	108.4 ± 11.4	0.670

Data are presented as mean ± standard deviation or median with interquartile range. SATa, area of subcutaneous adipose tissue; SATth, anterior subcutaneous fat thickness; VATa, area of visceral adipose tissue; PRAT, perirenal adipose tissue; BMI, body mass index; WC, waist circumference.

### Factors associated with changes in blood pressure after RDN

**Figure 3:**
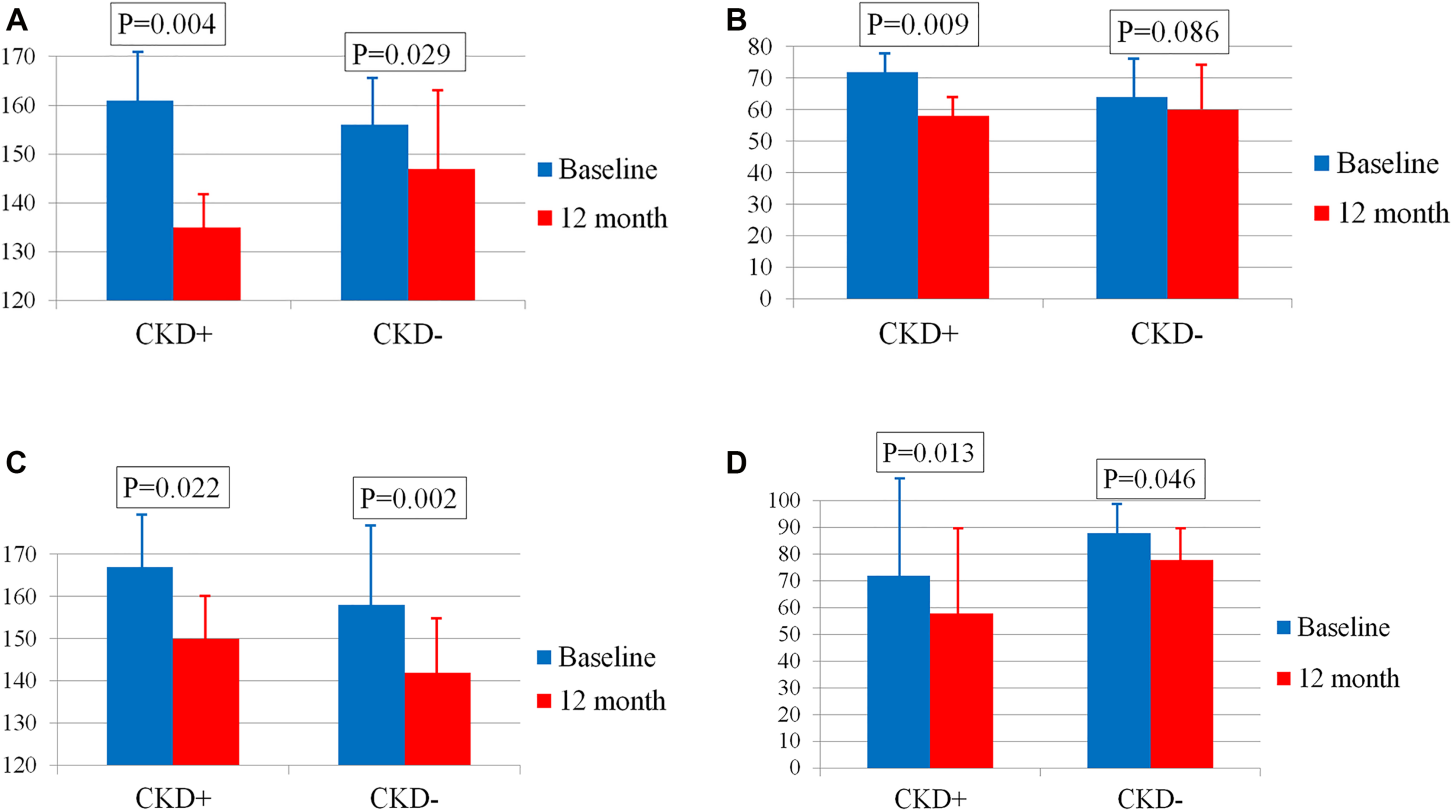
**(А)** Changes in SBP in men with and without CKD. **(B)** Changes in PP in men with and without CKD. **(C)** Changes in SBP in women with and without CKD. **(D)** Changes in PP in women with and without CKD. Bar graphs show the respective means ± standard deviations.

The decline in SBP and PP in men depends on the baseline eGFR. Thus, the reduction in SBP in male patients with CKD (*n* = 5) was almost three times greater than in those without CKD (*n* = 19) [−27[−27; −24] and −10[−17;0] mmHg, respectively, *p* = 0.004] (Figure 3A). The difference in PP reduction was even more pronounced and reached significance in patients with CKD only [−12[−14;−11] and −2[−10;1] mmHg, respectively, *p* = 0.023] (Figure 3B).

The decline in BP in women did not differ depending on the presence or absence of CKD. Thus, the BP reduction in female patients with CKD (*n* = 10) and without CKD (*n* = 23) was −19[−30; −4] and −12[−21; −1] mmHg, *p* = 0.652 for SBP (Figure 3C) and −9[−21; 0] and −6[−13; 0] mmHg, *p* = 0.695 for PP (Figure 3D) respectively.

**Figure 4:**
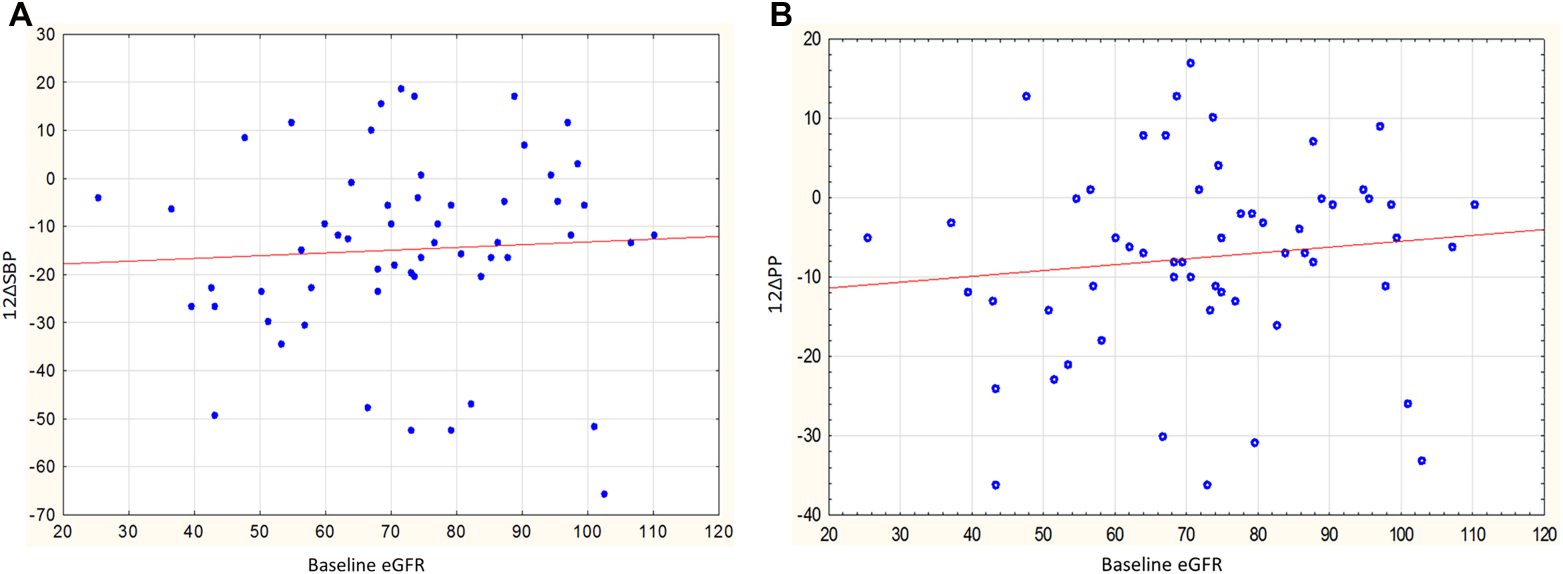
**(A)** Relationship between baseline eGFR and changes in SBP in men, *r* = 0.44, *p* = 0.034. **(B)** Relationship between baseline eGFR and changes in PP in men, *r* = 0.48, *p* = 0.017.

The decrease in SBP and PP in the male group was significantly more pronounced in individuals with a lower eGFR; the correlation coefficients of baseline eGFR with changes in SBP and PP were *r* = 0.44, *p* = 0.034 (Figure 4A) and *r* = 0.48, *p* = 0.017 (Figure 4B). A similar pattern was not observed in the female group: *r* = 0.01, *p* = 0.951 for SBP change, *r* = −0.02, *p* = 0.914 for PP change.

**Figure 5:**
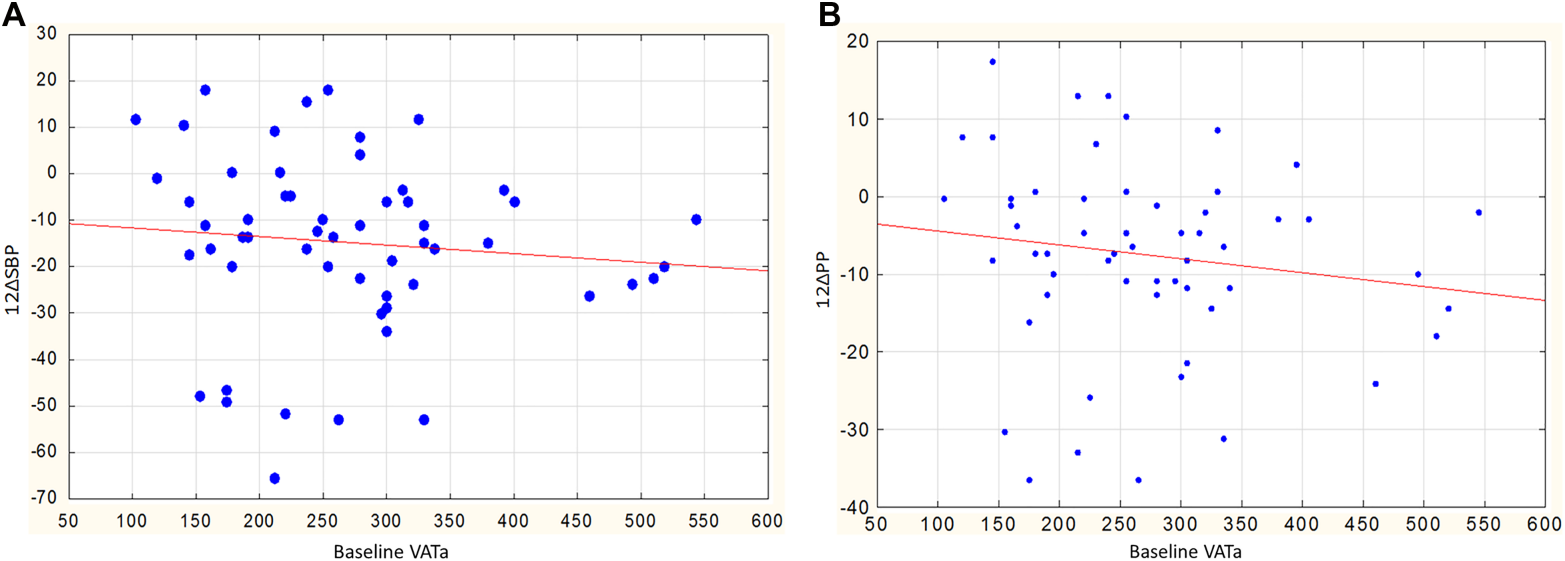
**(A)** Relationship between baseline VATa and changes in SBP in men, *r* = −0.44, *p* = 0.030. **(B)** Relationship between baseline VATa and changes in PP in men, *r* = −0.58, *p* = 0.003.

In men, the reduction in SBP and PP also depended on the parameters of visceral obesity. This is reflected in the inverse relationship between the reduction in SBP and PP and initial VATa (*r* = −0.44, *p* = 0.030 and *r* = −0.58, *p* = 0.003, Figures 5A, B, respectively). No similar relationships were observed in women: the correlation coefficients between the reduction in SBP and PP and the initial VATa were *r* = −0.08, *p* = 0.649, and *r* = −0.03, *p* = 0.877, respectively.

**Figure 6:**
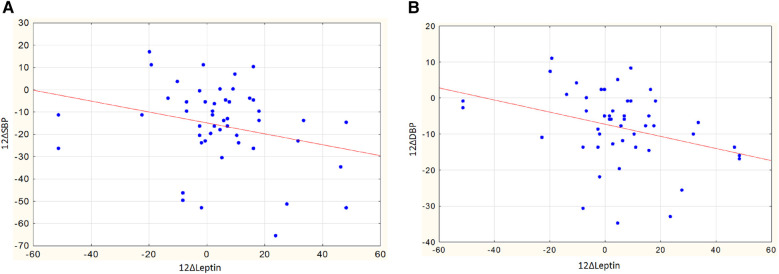
**(A)** Assotiations of changes in leptin and changes in SBP in women, *r* = −0.47, *p* = 0.012. **(B)** Assotiations of changes in leptin and changes in DBP in women, *r* = −0.48, *p* = 0.010.

In women, a greater reduction in SBP was observed in those with higher body weight and larger WC: changes in SBP showed an inverse correlation with baseline weight (*r* = −0.35, *p* = 0.047) and WC (*r* = −0.38, *p* = 0.029). Interestingly, in women, the decrease in SBP and DBP was also associated with an increase in leptin levels after RDN. The correlation coefficients of leptin changes with a decrease in SBP and DBP were *r* = −0.47, *p* = 0.012 (Figure 6A) and *r* = −0.48, *p* = 0.010 (Figure 6B), respectively.

In men, no significant correlations were observed between leptin levels and blood pressure. The correlation coefficients for changes in leptin with decreases in SBP and DBP were *r* = 0.09, *p* = 0.696, and *r* = −0.16, *p* = 0.498, respectively.

## Discussion

### BP

Renal denervation significantly reduced blood pressure regardless of sex, as previously confirmed by other studies.

### Biomarkers

The vast majority of patients included in the study were obese and had abdominal obesity, which is naturally associated with hyperleptinemia. Obesity leads to leptin resistance in relation to the anorexigenic effect but not in relation to the sympathetic nervous system. It is likely that increased sympathetic outflow is one mechanism by which increased leptin may increase PB through both centrally mediated effects on the hypothalamus and local peripheral effects, including increased sympathetic activity in the kidneys (27). The mechanism of the increase in leptin following the intervention is not yet entirely clear, particularly in relation to the decrease in BP in the female group, although other authors have observed no changes in this indicator (28, 29). Since leptin has been shown to activate renal sympathetic nerve activity (30) and RDN decreases both efferent and afferent signaling from the kidneys, the pathophysiological mechanism of increased leptin may be an inadequate sympathetic response to the ever-increasing leptin concentrations. The greater the drop in BP (and thus the more effective the denervation), the more pronounced the effect of the negative feedback. This mechanism is observed specifically in women, probably due to a large proportion of adipose tissue in general and of SAT in particular, as leptin levels are more strongly associated with SAT volume (31). However, this needs further investigation.

The lack of significant dynamics in other adipocytokines is consistent with literature data (28), except for a few studies (29, 32).

The examined patients with RHT were predominantly characterized by initially elevated plasma levels of normetanephrine. The effects of the sympathetic nervous system on blood pressure are mediated via adrenergic receptors and neurotransmitters (mainly norepinephrine and epinephrine) (33). It is clear that plasma levels of metanephrines and normetanephrines are only a crude indicator of sympathetic nervous system activity. However, the fact that norepinephrine is mainly released from the nerve terminals, while epinephrine is released from the adrenal medulla, makes the plasma level of normetanephrine more specific in this respect, which is also confirmed by its decrease after RDN in the female group. Ezzahti et al. (34) also observed a decrease in norepinephrine levels 6 and 12 months after RDN in contrast to adrenaline, although data from a large meta-analysis suggest that there are predominantly no changes in these markers (35). It is also necessary to consider the use of different analytical techniques. In the study on renal norepinephrine (for which porcine renal cortex tissue was used for measurement), RDN reduced it, and the effect was more pronounced when RDN was performed in branches of the renal artery closer to the kidney (36).

Undoubtedly, sex differences may influence the catecholamine system in some way, which is currently being demonstrated in other areas of medicine (37, 38) and may become the subject of further research.

### eGFR and CKD

RDN is a highly effective treatment for RHT. However, the potential of this method remains unexplored, as approximately one-third of patients do not respond to treatment (39). The efficacy of invasive treatment likely depends on the completeness of denervation, but routine assessment of the extent of renal sympathetic nerve damage during the procedure in humans remains elusive. Therefore, to improve the efficacy of renal denervation, it is advisable to select the most appropriate candidates.

There is a strong pathophysiologic rationale for RDN in the CKD population. There is evidence that overactivity of the sympathetic nervous system plays a role in the development of HT and in the development and progression of CKD. It is known that it is very difficult to control BP in patients with CKD. The results of the German Chronic Kidney Disease (GCKD) study show that <50% of CKD patients were controlled, and half of them met the criteria for RHT (40).

However, the safety of RDN in CKD patients needed to be evaluated first. According to the literature, the rate of decline in eGFR after RDN in this group is almost physiologic. For example, in the study by Hering D. et al., no progression of CKD was observed over 24 months after RDN, while one year before the procedure in this group, the decrease in eGFR was −3.41 ± 1.64 ml/min/1.73 m^2^ (41). According to our own 3-year observational data, the decline in eGFR in diabetic patients with RHT was almost physiologic (8). Long-term data from the Global SYMPLICITY Registry, in which 21 percent of patients had a baseline eGFR <60 ml/min/1.73 m^2^, show a decrease in renal function of 3.7 ml/min/1.73 m^2^ in patients with CKD and 7.1 ml/min/1.73 m^2^ in patients without CKD, and a similar BP-lowering effect (42).

Our study demonstrates not only the safety of RDN in patients with reduced GFR, but also its greater antihypertensive efficacy in the male half. The dependence of SBP and PP lowering on initial eGFR can be explained by more pronounced sympathetic overactivity in patients with a combination of HT and CKD (43), implying that the use of RDN in this group is more pathophysiologically justified and, therefore, more effective.

The reduction in eGFR in women without CKD is also consistent with the results of the Global SYMPLICITY Registry, where the most significant reduction in eGFR was observed in the first year of follow-up, similar to our three-year follow-up findings (44). The decrease in eGFR in the first year after the intervention could be functional due to reduced perfusion pressure due to BP reduction (45).

As for sex differences, we know that they exist naturally (46). Many studies show that the female sex is a protective factor for the progression of many kidney diseases. The European QUALity Study on treatment in advanced CKD (EQUAL) study found that kidney function declines faster in men with advanced CKD than in women (47). Unfortunately, the influence of sex on the development and progression of kidney disease remains poorly understood. The influence of sex hormones on the pathophysiology of sex differences in kidney damage and fibrosis ranges from metabolic processes to epigenetic programming (48). This currently requires further investigation.

### Adipose tissue

Conflicting data on the efficacy of RDN in obese patients may be due to the heterogeneity of adipose tissue in individuals with comparable BMI, which also varies widely by sex (49). To understand that it is VAT that plays a leading role in the development of a pathologic pattern in obesity, a more accurate quantitative assessment of visceral fat depots is needed. For this reason, accurate, precise, and reliable tools for segmenting and quantifying the distribution of adipose tissue in the body using non-invasive imaging such as CT and MRI have been explored in recent years. As there is currently no gold standard for measuring different fat depots, we have opted for what we believe is the simplest and most reproducible method for quantifying visceral and subcutaneous fat as well as perirenal fat.

With a more precise quantitative assessment of VAT, we found a correlation between the antihypertensive effect of RDN and VATa, but only in men. This can be explained primarily by the fact that people with a lot of visceral fat have a higher sympathetic tone (50), which largely determines the development of HT (33), and accordingly, the sympatholytic procedure is more effective in such cases. The greater proportion of VAT in men is likely responsible for the presence of such dependence. This is supported by the previously demonstrated relationship between muscle sympathetic nerve activity and the extent of abdominal visceral adipose tissue in men (51) and is not inconsistent with the data on the relationship between visceral obesity and the development of CKD (52). Both visceral obesity and CKD were associated with a greater reduction in BP in men in our study.

However, in women, the fall in BP after RDN also depended on the severity of abdominal obesity (the WC), confirming, on the one hand, the relationship between the RDN effect and the severity of visceral obesity, as WC is predominantly associated with visceral fat (53), and on the other hand, suggesting that it is less important to use MRI to select optimal candidates for invasive treatment of RHT in women. The importance of abdominal obesity in selecting patients more responsive to RDN was previously shown in the RADIANCE-HTN SOLO trial using ultrasonography (54), regardless of sex.

A decrease in PRAT was another positive effect of the intervention in the female group. This was discovered for the first time, was not previously found in the literature, and undoubtedly requires further investigation.

Thus, numerous studies on the efficacy of RDN show conflicting data on sex-specific response characteristics (55, 56). However, the data on response in obese individuals are more robust and also have a pathophysiologic basis. Sex-specific differences in the distribution of adipose tissue and the pathophysiological importance of specific fat depots are evident (57). The CKD-EPI formula also contains sex-specific differences. Therefore, we believe different parameters can also be used when selecting men and women for the RDN procedure, which is confirmed by our results. At the same time, further investigation of VAT parameters compared to the effectiveness of RDN could provide a more universal indicator for predicting its effectiveness.

## Conclusions

RDN is associated with a pronounced antihypertensive effect, the magnitude of which in both sexes depends on signs of visceral obesity, and in men, it also depends on the presence of CKD. Renoprotective effects of RDN in men are obtained regardless of the initial functional status of the kidneys, while in women, it was observed only in individuals with CKD. Additional beneficial effects of RDN in women include a decrease in serum levels of normetanephrines and a reduction in PRAT size.

### Limitations

This study had several limitations. The small sample size may not have been meaningful enough to detect some differences between groups, e.g., in VATa. In addition, our sample was heterogeneous and included patients with and without obesity, diabetes mellitus, CKD, and other diseases. Primary hyperaldosteronism (PA) was excluded based on the results of blood potassium, aldosterone, renin, and aldosterone to renin ratio, 24 h urine collection for sodium and potassium, and MRI of the adrenal glands. However, the guidelines require a more complex algorithm, including specific blood sampling requirements, sodium loading tests, captopril and fludrocortisone tests, adrenal vein sampling, and discontinuation of a number of antihypertensive drugs if possible (58). In this regard, a retrospective assessment of patients with RHT is planned for PA, which, according to the REQUIRE study, occurred in 32.4% (59). According to the authors of this study, the high frequency of PA could to some extent explain the lack of a significant difference in BP reduction between RDN and the sham procedure. Indeed, Miyajima et al. found that MSNA was decreased in patients with PA compared with patients with essential hypertension (*p* < 0.01) and normotensive subjects (*p* < 0.1) (60). Therefore, performing RDN in patients with PA is apparently not pathophysiologically justified.

The patients had stable but non-standardized drug therapy, about which we obtained information by interview. Therefore, it was not possible in this study to assess whether and how the response of BP after RDN depends on concomitant antihypertensive medication. Perhaps future studies will help to derive a “success formula” for predicting the blood pressure-lowering effect of renal denervation in patients with visceral obesity and CKD.

## Data Availability

The raw data supporting the conclusions of this article will be made available by the authors, without undue reservation.
